# Assessing the Power of the HEXACO to Predict Professional Burnout Among Catholic Priests in Italy

**DOI:** 10.1007/s10943-024-02202-5

**Published:** 2024-12-31

**Authors:** Giuseppe Crea, Leslie J. Francis, Ursula McKenna

**Affiliations:** 1 Salesian Pontifical University, Rome, Italy; 2 Centre for Educational Development, Appraisal and Research (CEDAR), The University of Warwick, Coventry, UK; 3 World Religions and Education Research Unit, Bishop Grosseteste University, Lincoln, UK

**Keywords:** Balanced affect, Francis Burnout Inventory, Catholic priests, Italy, HEXACO

## Abstract

This study tests the application of the HEXACO among Catholic priests and the power of this six factor model of personality to predict scores on the Francis Burnout Inventory among priests. Data provided by 264 priests serving in Italy lead to two conclusions. In this population three of the six scales of the HEXACO failed to display adequate levels of internal consistency reliability (emotionality, agreeableness, openness to experience). High scores of extraversion and conscientiousness predicted higher scores of satisfaction in ministry. Low scores of extraversion, conscientiousness, *and* honesty and humility predicted higher scores of emotional exhaustion in ministry. The role of the honesty and humility factor in predicting negative affect but not positive affect supports the balanced affect model of professional burnout that views positive and negative affect as partly independent systems.

## Introduction

Research designed to explore predictors of individual differences in work-related psychological wellbeing and professional burnout among clergy has drawn attention to the importance of internal personality factors above external contextual or situational factors. This body of research has drawn on the models of personality proposed by the Eysenckian three major dimensions (Eysenck & Eysenck, 1975, 1991), by the big five factors (Costa & McCrae, 1985), and the Jungian notion of psychological type as operationalised by the Myers-Briggs Type Indicator (Myers & McCaulley, 1985), the Keirsey Temperament Sorter (Keirsey & Bates, 1978), and the Francis Psychological Type Scales (Francis, 2005; Francis, Laycock, & Brewster, 2017).

Studies on clergy burnout employing the three major dimensions model alongside the Maslach Burnout Inventory (Maslach & Jackson, 1986) have been reported by Rutledge and Francis (2004), Francis, Louden, and Rutledge (2004), and Francis and Turton (2004a, 2004b). For example, in a study of 1071 Anglican clergymen in England, Rutledge and Francis (2004) found that: depersonalisation was positively associated with neuroticism (*β* = .32), positively associated with psychoticism (*β* = .24), and negatively associated with extraversion (*β* =  − .14); emotional exhaustion was positively associated with neuroticism (*β* = .46), positively associated with psychoticism (*β* = .13), and negatively associated with extraversion (*β* =  − .15); personal accomplishment was positively associated with extraversion (*β* = .42), negatively associated with neuroticism (*β* =  − .22), and negatively associated with psychoticism (*β* =  − .06).

Studies on clergy burnout employing the big five factor model alongside the Maslach Burnout Inventory (Maslach & Jackson, 1986) have been reported by Miner (2007a, 2007b), Joseph et al. (2011), and Stephens (2020). For example, in a study among 603 Protestant clergy in the USA, Stephens (2020) found that: depersonalisation was positively associated with neuroticism (*β* = .45), negatively associated with extraversion (*β* =  − .07), negatively associated with agreeableness (*β* =  − .27), and independent of openness and conscientiousness; emotional exhaustion was positively associated with neuroticism (*β* = .60), positively associated with conscientiousness (*β* = .07), negatively associated with extraversion (*β* =  − .22), and independent of openness and agreeableness; personal accomplishment was negatively associated with neuroticism (*β* =  − .23), positively asssociated with openness (*β* = .24), positively associated with conscientiousness (*β* = .18), positively associated with extraversion (*β* = .28), and independent of agreeableness.

Studies on clergy burnout employing the psychological type model alongside the Francis Burnout Inventory (Francis, Kaldor, et al., 2005) have been reported by Francis, Wulff, and Robbins (2008), Francis, Robbins, et al. (2009), and Francis and Crea (2015). For example, in a study of 748 clergy serving within the Presbyterian Church (USA), Francis, Wulff, and Robbins (2008) found that clergy who prefer extraversion (rather than introversion) recorded higher scores of satisfaction in ministry and lower scores of emotional exhaustion in ministry.

During the past two decades a new, six factor model of personality has emerged into prominence, known as the HEXACO. The aim of the present paper is to examine and critique this new model, and then to explore its potential as a predictor of individual differences in work-related psychological wellbeing and professional burnout in a new study conducted among Catholic priests in Italy.

### Introducing the HEXACO Six Factor Model of Personality

Building on the established Big Five Factor model of personality as exemplified by Costa and McCrae (1985), in a series of studies Lee and Ashton introduced and tested the six factor model known as the HEXACO (see Ashton & Lee, 2005, 2007, 2008, 2009, 2010; Ashton et al., 2004a, 2004b; Lee & Ashton, 2006, 2008, 2012a, 2012b). The core novelty and innovation of the HEXACO reside in the introduction of the sixth factor of personality, identified as ‘The H factor’. The two words that characterise high scores on this factor are honesty and humility. The key personality-descriptive adjectives observed in lexical studies that define higher scores on the H factor are: sincere, honest, faithful, loyal, modest, unassuming, fair minded, and ethical. Two example items in the instrument designed to assess *honesty and humility* are:

- I wouldn’t use flattery to get a raise or promotion at work, even if I thought it would succeed.
- I wouldn’t pretend to like someone just to get the person to do favours for me.

The key personality-descriptive adjectives that define low scores on the H factor are: sly, deceitful, greedy, pretentious, hypocritical, boastful, pompous, conceited, and self-centred. Two examples of reverse-coded items in the instrument designed to assess honesty and humility are:

- I think I am entitled to more respect than the average person is.
- I want people to know that I am an important person of high station.

While the other five factors of personality assessed by the HEXACO have been assigned the same established names associated with the big five factor model as promoted by Costa and McCrae (1985), the operationalisation of these factors is distinctive in ways that do not facilitate strict comparability with data generated by original measures of the big five factors. This is especially the case in respect of emotionality and agreeableness (Ashton et al., 2014).

High scores on the *emotionality* factor assessed by the HEXACO are characterised by the following key personality-descriptive adjectives: emotional, oversensitive, sentimental, fearful, anxious, nervous, vulnerable, and clingy. Two example items in the instrument designed to assess emotionality are:

- I would feel afraid if I had to travel in bad weather conditions.
- When I suffer from a painful experience, I need someone to make me feel comfortable.

Key personality-descriptive adjectives that define low scores on the emotional factor are: tough, fearless, unemotional, independent, self-assured, unfeeling, and incentive. Two examples of reverse-coded items are:

- I can handle difficult situations without needing emotional support from anyone else.
- Even in an emergency I wouldn’t feel like panicking.

High scores on the *agreeableness* factor assessed by the HEXACO are characterised by the following key personality-descriptive adjectives: patient, tolerant, peaceful, mild, agreeable, lenient, gentle, and forgiving. Two items in the instrument designed to assess agreeableness are:

- I rarely hold a grudge, even against people who have badly wronged me.
- Even when people make a lot of mistakes, I rarely say anything negative.

Key personality-descriptive adjectives that define low scores on the agreeableness factor are: ill-tempered, quarrelsome, stubborn, choleric, temperamental, headstrong, and blunt. Two examples of reverse-coded items are:

- When people tell me that I’m wrong, my first reaction is to argue with them.
- People think of me as someone who has a quick temper.

High scores on the *conscientiousness* factor assessed by the HEXACO are characterised by the following key personality-descriptive adjectives: organised, self-disciplined, hard-working, efficient, careful, thorough, precise, and perfectionist. Two items in the instrument designed to assess conscientiousness are:

- I often push myself very hard when trying to achieve a goal.
- People often call me a perfectionist.

Key personality-descriptive adjectives that define low scores on the conscientiousness factor are: sloppy, negligent, reckless, lazy, irresponsible, absent-minded, and messy. Two examples of reverse-coded items are:

- I do only the minimum amount of work needed to get by.
- I make a lot of mistakes because I don’t think before I act.

High scores on the *openness to experience* factor assessed by the HEXACO are characterised by the following key personality-descriptive adjectives: intellectual, creative, unconventional, imaginative, innovative, complex, deep, inquisitive, and philosophical. Two items in the instrument designed to assess openness to experience are:

- I like people who have unconventional views.
- I would enjoy creating a work of art, such as a novel, a song, or a painting.

Key personality-descriptive adjectives that define low scores on the openness to experience factor are: shallow, simple, unimaginative, conventional, and closed-minded. Two examples of reverse-coded items are:

- I think that paying attention to radical ideas is a waste of time.
- I would be quite bored by a visit to an art gallery.

High scores on the *extraversion* factor assessed by the HEXACO are characterised by the following key personality-descriptive adjectives: outgoing, lively, extraverted, sociable, talkative, cheerful, active, vocal, and confident. Two items in the instrument designed to assess extraversion are:

- I feel reasonably satisfied with myself overall.
- On most days, I feel cheerful and optimistic.

Key personality descriptive adjectives that define low scores on the extraversion scale are: shy, passive, withdrawn, introverted, quiet, reserved, inhibited, and gloomy. Two examples of reverse-coded items are:

- I feel that I am an unpopular person.
- I sometimes feel that I am a worthless person.

The foregoing analysis of the roots of the HEXACO six factor model of personality is in lexical studies and the example items for the six scales illustrate the potential distinctive strength of this model, and also its potential weaknesses within the broader context of studies within the tradition of personality and individual differences. The core conceptual distinction concerns what characteristics may best differentiate the construct of personality from related constructs like character and psychopathology. In serious critique within this conceptual tradition, Lloyd (2015) has previously cautioned the potential confusion between personality and character in established conceptualisations and operationalisations of the big five factor model of personality, especially in the sense that low scores on these measures were characterised by undesirable features; for example, the opposite of agreeableness emerges as disagreeableness. The HEXACO is perhaps even more vulnerable to this criticism.

The long established Eysenckian three-dimensional model of personality consciously conceptualised a continuum between normal personality and psychopathology as recognised in the high scoring poles of the two scales known as neuroticism and psychoticism (Eysenck & Eysenck, 1975, 1976). However, the operationalised form of these measures was based on items that noted subclinical precursors of these pathologies, an approach that was particularly successful in the neuroticism scale and never quite resolved in the psychoticism scale, as reflected in the low endorsement of the more extreme items and the poor level of internal consistency reliability (Francis, Brown, and Philipchalk, 1992). The item selection for the HEXACO is perhaps more vulnerable to the criticism of loading the assessment of individual differences in normal personality with indicators more generally associated with psychopathologies.

In an early study, Lee and Ashton (2005) specifically anchored the HEXACO model of personality against psychopathy as measured by Levenson et al. (1995), Machiavellianism as measured by Christie and Geis (1970), and narcissism as measured by Raskin and Terry (1988). The associations with honesty and humility were particularly strong: psychopathy, *r* =  − .72; Machiavellianism, *r* =  − .57; narcissism, *r* =  − .53. Two subsequent studies, using the Dirty Dozen measure of the Dark Triad (Jonason & Webster, 2010) confirmed these associations. Jonason and McCain (2012) reported the following correlations with honesty and humility: psychopathy, *r* =  − .38; Machiavellianism, *r* =  − .52; narcissism, *r* =  − .52. Aghababaei et al. (2014) reported the following correlations with honesty and humility: psychopathy, *r* =  − .36; Machiavellianism, *r* =  − .58; narcissism, *r* =  − .36. The first of these studies also reported correlations in excess of *r* =  − .20: between psychopathy and extraversion (*r* =  − .23), agreeableness (*r* =  − .29), consciousness (*r* =  − .33), and emotionality (*r* =  − .27); and between Machiavellianism and agreeableness (*r* =  − .27), and consciousness (*r* =  − .35); and between narcissism and agreeableness (*r* =  − .21).

A further and somewhat different problem with the HEXACO emerges in relation to the conceptualisation of extraversion and introversion, particularly as viewed through the lens of the Eysenckian refinement of this continuum. A core insight from the Eysenckian model concerns the clear orthogonality between extraversion and neuroticism. In the Eysenckian system extraversion and emotional stability, introversion and emotional instability are not aligned (Eysenck & Eysenck, 1975, 1976). In this model it is reasonable to speak of neurotic extraverts and stable introverts. A similar point was made by the earlier work of Cattell et al. (1970). The confusion of these constructs within the HEXACO may be a retrograde step.

### Clergy Work-Related Psychological Wellbeing and Professional Burnout

The current literature on the predictors of individual differences in work-related psychological wellbeing and professional burnout among clergy has been largely driven by two different conceptualisations and operationalisations of burnout. The longer established measure within the literature is the Maslach Burnout Inventory (MBI; Maslach & Jackson, 1986). The MBI assesses three constructs, styled emotional exhaustion, depersonalisation, and personal accomplishment. The underlying theory proposes a sequential progression among those engaged in caring or people-facing professions, beginning with emotional exhaustion. Experienced emotional exhaustion leads to disengagement with clients as reflected in depersonalisation. Depersonalisation leads to dissatisfaction or disappointment among clients who in turn withdrew satisfaction in a way that practitioners recognise as eroding their sense of personal accomplishment.

Examples of clergy studies employing the MBI include work reported by Warner and Carter (1984), Crea (1994), Strümpfer and Bands (1996), Rodgerson and Piedmont (1998), Stanton-Rich and Iso-Ahola (1998), Virginia (1998), Evers and Tomic (2003), Golden et al. (2004), Raj and Dean (2005), Miner (2007a, 2007b), Doolittle (2007, 2010), Chandler (2009), Joseph et al. (2010), Buys and Rothmann (2010), Parker and Martin (2011), Joseph et al. (2011), Rossetti (2011), Küçüksüleymanoğlu (2013), Rossetti and Rhoades (2013), Herrera et al. (2014), Proeschold-Bell et al. (2014), Crea and Francis (2015), Adams et al. (2017), Büssing et al. (2017), Vicente-Galindo et al. (2017), Chan and Chen (2019), Dias (2019), Case et al. (2020), Muasa et al. (2021), Malcolm et al. (2022), Proeschold-Bell et al. (2022), and Sanagiotto (2024).

In the 1990s Rutledge and colleagues challenged the appropriateness of items in the MBI for use among clergy and sought permission from the test publishers to develop a revised form of the MBI adapted for the clerical profession. This revised measure under licence and at a cost has been tested in a series of studies, including Francis and Rutledge (2000), Kay (2000), Francis, Louden, and Rutledge (2004), Francis and Turton (2004a, 2004b), Hills et al. (2004), Randall (2004, 2007), Rutledge (2006), Turton and Francis (2007), Francis, Turton, and Louden (2007), Francis, Robbins, et al. (2010), and Miller-Clarkson (2013).

The more recent measure employed within the literature on individual differences in work-related psychological wellbeing and professional burnout among clergy, the Francis Burnout Inventory (FBI; Francis, Kaldor, 2005) was developed specifically with the clerical profession in view. The FBI assesses two constructs, styled emotional exhaustion and satisfaction in ministry. The underlying theory is rooted in the classic work of Bradburn (1969) concerning the model of balanced affect. This model suggests that positive affect and negative affect operate as partially separate systems. In the case of clergy it is reasonable to posit individuals who record both high levels of negative affect (emotional exhaustion) together with high levels of positive affect (satisfaction in ministry). The model of balanced affect suggests that high levels of positive affect can mitigate the consequences of high levels of negative affect.

A series of studies has now validated the balanced affect model of clergy work-related psychological wellbeing, including work reported by Francis, Village, et al. (2011) among 744 clergy in The Presbyterian Church USA, Francis, Laycock, and Brewster (2017) among 658 clergy in the Church of England, Francis, Laycock, and Crea (2017) among 155 priests in the Roman Catholic Church in Italy, Francis, Crea, and Laycock (2017) among 95 priests and 61 religious sisters in the Roman Catholic Church in Italy, Village et al. (2018) among 358 Anglican clergy in the Church in Wales, Francis, Laycock, and Ratter (2019) among 99 Anglican clergy in England, Francis et al. (2021) among 287 priests in the Roman Catholic Church in Italy, and Francis, Village, and Haley (2023) among 803 Methodist ministers serving in Great Britain.

These validation studies have demonstrated how positive affect works to mitigate the detrimental effects of negative affect. This finding then carries significant implications for managing clergy work-related psychological wellbeing. While it may be difficult to remove the causes of negative affect generated by pastoral ministry, it may be easier to design intentional ways of enhancing positive affect.

### Research Questions

Against this background the aim of the present study is to invite Catholic priests in Italy to complete the HEXACO alongside the FBI in order to address the following two primary research questions concerning:

- The psychometric properties of the HEXACO among clergy
- The power of the HEXACO to predict individual differences within the balanced affect model of clergy work-related psychological wellbeing and professional burnout.

## Method

### Procedure

A team of trainee psychologists working within the Pontifical Salesian University in Rome employed the snowball sampling technique to recruit a diverse sample of practising Catholic priests in Italy to participate in the project. Participation in completing the survey was voluntary and unrewarded, and responses were confidential and anonymous.

### Participants

Fully completed surveys were returned by 264 priests, ranging in age from 25 to 84 years: 130 were under the age of forty, 73 were in their forties, 36 were in their fifties, 13 were in their sixties, 11 were aged seventy or over, and one failed to record his age; 150 served as diocesan priests and 114 as religious priests; in terms of nationality, 117 were Italian and 147 were not.

### Measures

*Professional burnout* was assessed by the Francis Burnout Inventory (FBI; Francis, Kaldor, et al., 2005). This 22-item instrument comprises the 11-item Scale of Emotional Exhaustion in Ministry (SEEM) and the 11-item Satisfaction in Ministry Scale (SIMS). Each item was assessed on a five-point scale: agree strongly (5), agree (4), not certain (3), disagree (2), and disagree strongly (1). For the Italian translation of this measure, Francis and Crea (2015) reported alpha coefficients of .79 for SIMS and .81 for SEEM.

*Personality* was assessed by the 60-item HEXACO (Lee & Ashton, 2012b). This instrument comprises six 10-item measures of honesty and humility, emotionality, extraversion, agreeableness, conscientiousness, and openness to experience. Each item was assessed on a five-point scale: agree strongly (5), agree (4), neutral (3), disagree (2), and disagree strongly (1). For the Italian translation of this measure, Baiocco et al. (2017) reported alpha coefficients of .71 for honesty and humility, .72 for emotionality, .76 for extraversion, .74 for agreeableness, .71 for conscientiousness, and .70 for openness to experience.

### Data Analysis

The data were analysed by means of the SPSS statistical package, using the reliability, correlation, and regression routines.

## Results and Discussion

The first step in data analysis explored the psychometric properties of the six scales proposed by the HEXACO among Catholic priests, in terms of the alpha coefficient of internal consistency reliability (Cronbach, 1951), the correlations between the individual items and the sum of the other nine items, and the item endorsement in items of the sum of the agree and agree strongly responses. Each of the six scales will be reviewed in turn (see Appendix 1).

*Honesty and humility* achieved an adequate level of internal consistency reliability (*α* = .71) and each item correlated relatively well with the sum of the other nine items (range .28–.44). The low item endorsement of the six reverse-coded items indicated a poor level of item discrimination, some of which can be explained by the characteristics of pathology underpinning those items. For example, just 6% of priests agree that if they want something from someone, they will laugh at that person’s worst jokes (an idea reflecting the manipulation component of Machiavellianism). Just 11% of priests agree that they want people to know that they are an important person of high status (an idea reflecting the grandiosity component of narcissism). Very few priests agree that if they knew that they could not be caught, they would be willing to steal a million dollars (4%), or that they would be tempted to use counterfeit money, if they were sure they could get away with it (3%) (two ideas reflecting the antisocial behaviour component of psychopathy).

*Emotionality* failed to reach an adequate level of internal consistency reliability (*α* = .56). This suggests that the range of issues clustered within the ten items fail to function as a unidimensional construct among Catholic priests.

*Extraversion* reached a recognised threshold for an acceptable level of internal consistency reliability (*α* = .66). This construct combined the social dimension of extraversion (50% of priests agreed that in social situations, they are usually the one who makes the first move), with good self-esteem (79% of priests felt reasonably satisfied with themselves overall), and with personal optimism (77% of priests felt cheerful and optimistic on most days).

*Agreeableness* failed to reach an adequate level of internal consistency reliability (*α* = .46). This suggests that the range of issues clustered together within the ten items fail to function as a unidimensional construct among Catholic priests.

*Conscientiousness* achieved an adequate level of internal consistency reliability (*α* = .73). The item endorsement suggests a high level of conscientiousness among Catholic priests: 87% often push themselves very hard when trying to achieve a goal; 84% always try to be accurate in their work, even at the expense of time; and 75% plan ahead and organise things, to avoid scrambling at the last minute.

*Openness to experience* only just failed to reach an adequate level of internal consistency reliability (*α* = .63). This scale was damaged by two items that achieved poor correlations with the sum of the other nine items (I think that paying attention to radical ideas is a waste of time, and I like people who have unconventional views). The endorsement of positively voiced items suggest quite a high level of openness to experience among Catholic priests: 71% are interested in learning about the history and politics of other countries; 71% would enjoy creating a work of art, such as a novel, a song, or a painting; 66% would like to attend a classical music concert if they had the opportunity.

The second step in data analysis explored the correlations among the six personality factors (see Table 1). Only one of the six factors displays clear independence of the other five namely the factor of emotionality. Both extraversion and honesty and humility are strongly correlated with the four other factors. These high correlations also call into question the six-factor model of the HEXACO.

**Table 1:** Correlations among the six personality factors

	H	Op	Co	Ag	Ex
Emotionality (Em)	− .11	− .09	− .03	− .10	− .12
Extraversion (Ex	.41^***^	.42^***^	.35^***^	.23^***^	
Agreeableness (Ag)	.27^***^	.14^*^	.23^***^		
Conscientiousness (Co)	.40^***^	.41^***^			
Openness (Op)	.29^***^				

*H* honesty and humility

*p < .05; ***p < .001

The third step in data analysis explored the psychometric properties of the two scales proposed by the FBI in terms of the alpha coefficient of internal consistency reliability (Cronbach, 1951), the correlations between the individual items and the sum of the other ten items, and the item endorsement in terms of the sum of the agree and agree strongly responses (see Appendix 2).

*Scale of Emotional Exhaustion in Ministry* achieved a satisfactory level of internal consistency reliability (*α* = .82). The item rest of scale correlations were all in the range of .35–.62, showing homogeneity among the items. The level of item endorsement suggests that up to one in seven of these priests recognised signs of burnout in their experience: 16% reported fatigue and irritation as part of their daily experience; 15% reported feeling drained by fulfilling their ministry role; 14% were less patient with those among whom they minister than they used to be.

*Satisfaction in Ministry Scale* achieved a satisfactory level of internal consistency reliability (*α* = .85). The item rest of scale correlations were all in the range of .42–.63, showing homogeneity among the items. The level of item endorsement suggests that the majority of these priests recognised high levels of work-related satisfaction: 89% were really glad that they had entered the ministry; 86% gain a lot of personal satisfaction from fulfilling their ministry roles; 82% feel very positive about their current ministry.

The fourth step in data analysis explored the bivariate correlations between each of the six factors of the HEXACO and the two scales of the FBI (see Table 2). These correlations show that only one of the six HEXACO scales was unrelated to individual differences in both positive affect and negative affect, namely the emotionality factor. In one sense this is strange since previous research employing either the Eysenckian three-dimensional model or the big five factor model has identified emotionality or neuroticism as a key predictor of individual differences in both positive and negative affect. Here is clear evidence that what the HEXACO is measuring under the name of emotionality is discontinuous with previous personality measures employing that descriptor.

**Table 2:** Correlations between the Francis burnout inventory and the HEXACO model

	SEEM	SIMS
Honesty and humility	− .53^***^	.38^***^
Emotionality	.09	− .07
Extraversion	− .46^***^	.51^***^
Agreeableness	− .26^***^	.21^***^
Conscientiousness	− .44^***^	.45^***^
Openness to experience	− .25^***^	.40^***^

***p < .001

Given that five of the HEXACO factors are each individually correlated with both positive affect and negative affect, and given that these five factors are themselves heavily intercorrelated, the fifth step in data analysis employed multiple regression to clarify the unique contribution made by these factors. Table 3 demonstrates that in terms of positive affect (satisfaction in ministry) the key predictors are extraversion (*β* = .32) and conscientiousness (*β* = .23), with some extra prediction provided by openness (*β* = .14). When these three factors are taken into account, honesty and humility offers no additional predictive power. Table 4 demonstrates that in terms of negative affect (emotional exhaustion in ministry) the key predictors are extraversion (*β* =  −.25), conscientiousness (*β* =  − .23), *and* honesty and humility (*β* =  − .33). The finding that honesty and humility is the main predictor of emotional exhaustion in ministry, but is not an active predictor of satisfaction in ministry adds further weight to the theory that positive affect and negative affect operate as partly independent systems and are not the mirror image of each other.

**Table 3:** Regression models on satisfaction in ministry scale

	Model 1	Model 2	Model 3	Model 4	Model 5	Model 6
Extraversion	.51^***^	.51^***^	.40^***^	.36^***^	.35^***^	.32^***^
Emotionality		− .01	− .01	− .00	.00	.01
Conscientiousness			.31^***^	.27^***^	.26^***^	.23^***^
Openness				.14^*^	.14^*^	.14^*^
Agreeableness					.05	.03
Honesty and humility						.11
Δ	.26^***^	.00	.09^***^	.02^*^	.00	.01
*R*^*2*^	.26	.26	.34	.36	.36	.37

*p < .05; ***p <.001

**Table 4:** Regression models on emotional exhaustion in ministry scale

	Model 1	Model 2	Model 3	Model 4	Model 5	Model 6
Extraversion	− .46^***^	− .46^***^	− .35^***^	− .36^***^	− .34	− .25^***^
Emotionality		.04	.04	.04	.03	.02
Conscientiousness			− .32^***^	− .33^***^	− .31^***^	− .23^***^
Openness				.04	.04	.06
Agreeableness					− .12^*^	− .07
Honesty and humility						− .33^***^
Δ	.21^***^	.00	.09^***^	.00	.01^*^	.08^***^
*R*^*2*^	.21	.21	.31	.31	.32	.40

*p <.05; ***p < .001

## Conclusion

The present study was designed to address two primary research questions set within the individual differences approach to exploring the connections between personality and work-related psychological wellbeing and professional burnout among clergy, concerning:

- The psychometric properties of the HEXACO among clergy
- The power of the HEXACO to predict individual differences within the balanced affect model of clergy, work-related psychological wellbeing, and professional burnout.

A body of previous research using three different models of personality (the Eysenckian three major dimensions model, the big five factors model, and psychological type theory) had demonstrated the power of personality theory to predict individual differences in professional burnout and had identified extraversion and emotionality (neuroticism) as two stable predictors. Recognising the emergence of a new model of personality proposing six factors, the present study focused attention on the performance of the HEXACO among Catholic priests in Italy. These two primary research questions set by the present study were addressed employing data provided by 264 Catholic priests serving in Italy who completed the HEXACO together with the Francis Burnout Inventory.

Before engaging with the analysis of these new data conceptual investigation of the HEXACO had raised two important questions about this innovative measure that in turn lead to theoretical conclusions that nuance the interpretation of the data. The first of these two theoretical conclusions is that it would be a mistake to assume that simply because the HEXACO adopts the same names for five of its factors as adopted by the classic operationalisations of the big five factor model, as is the case, for example, by Costa and McCrae (1985), these HEXACO factors operationalise the same constructs. This caveat applies especially to the two factors held largely in common with the classic operationalisation of the big five factor model and the Eysenckian three-dimensional model, namely extraversion and neuroticism (emotionality). Both of these factors have been shown to be core to the prediction of individual differences in clergy wellbeing in the big five factor model and the three-dimensional model. Both of these factors have been nuanced in significantly different ways in the HEXACO model. In other words, findings cannot be read across these factors from one model to another.

The second of these two theoretical conclusions is that it would be a mistake to underestimate the extent to which the new factor proposed by the HEXACO, namely honesty and humility, is nuanced to reflect characteristics highly correlated with the dark triad of subclinical pathologies: Machiavellianism, narcissism, and psychopathy. This distinctive nuancing places the honesty and humility factor somewhat out of step with the general range of measures concerned with normal personality. Specifically within the area of individual differences, clergy work-related psychological wellbeing, and professional burnout, an earlier study reported by Francis and Crea (2021) has demonstrated the value of differentiation between the predictive power of individual differences in normal personality and the predictive power of the dark triad of subclinical pathologies.

Against this background, the first empirical research question concerned examining the psychometric properties of the HEXACO among clergy. Two of the six scales fell far short of the base level of acceptable internal consistency reliability (emotionality and agreeableness) and a third also missed the minimum threshold (openness to experience). Given that unreliable measures by definition cannot serve as valid measures, the first empirical conclusion is that these data do not commend the full HEXACO for further application in clergy studies.

The second empirical research question concerned examining the power of the HEXACO to predict individual differences within the balanced affect model of clergy work-related psychological wellbeing and professional burnout. The present study took the view that it was sensible to test all six measures in the regression model (the three that failed to reach an acceptable level of reliability as well as the three stronger measures). The outcome of the regression model excluded the three weak measures (emotionality, agreeableness, and openness to experience) and demonstrated that each of the three remaining measures had a significant part to play. The role of each of these three measures deserves comment, giving rise to three further empirical conclusions.

The second empirical conclusion concerns the role of the HEXACO extraversion scale in predicting individual differences in both positive affect (satisfaction in ministry) and negative affect (emotional exhaustion in ministry). This predictive power is consistent with the HEXACO conceptualisation of the extraversion scale as mapping the continuum from stable extraversion to neurotic introversion, thereby capturing part of the two best established predictors of individual differences in work-related psychological wellbeing, namely extraversion and neuroticism, although clearly missing out on the capacity for neurotic extraverts and stable introverts. While an effective predictor of both positive affect and negative affect, the HEXACO extraversion scale may be less efficient than the earlier instruments that differentiated between extraversion and neuroticism.

The third empirical conclusion concerns the role of the HEXACO conscientiousness scale in predicting individual differences in both positive affect (satisfaction in ministry) and negative affect (emotional exhaustion in ministry). This finding suggests that higher levels of burnout are associated with the following personality-descriptive adjectives: sloppy, negligent, reckless, lazy, irresponsible, absent-minded, and messy. Higher levels of burnout are associated with priests who only do the minimum amount of work needed to get by, or who make a lot of mistakes because they do not think before they act.

The fourth, and most important, conclusion concerns the role of the HEXACO honesty and humility scale in predicting individual differences in clergy work-related psychological wellbeing. This factor is of particular interest because it predicts individual differences in negative affect, but not in positive affect. That finding is important for two reasons. First, it is consistent with the findings of Francis and Crea (2021) who tested the impact of the dark triad of Machiavellianism, neuroticism, and psychopathy separately on the two scales of the Francis Burnout Inventory. That study also found that the dark triad impacted emotional exhaustion in ministry but not satisfaction in ministry. The tentative conclusions can therefore be drawn that the honesty and humility factor functions in the same way as the dark triad. Second, it is consistent with the theory underpinning the balanced affect approach to clergy work-related psychological wellbeing and professional burnout, namely that positive affect and negative affect operate as partly independent systems that may consequently be impacted by different predictor variables. The tentative conclusion can therefore be drawn that the honesty and humility factor offers further construct validation for the balanced affect theory operationalised by Francis Burnout Inventory.

The main limitations with the present study arise from only one group of clergy being included in the study (Catholic priests serving in Italy) and from this group of clergy having been recruited by snowball sampling. Nonetheless, as the first study to have introduced the HEXACO to the field of clergy wellbeing research, the study has been successful in drawing attention to the weaknesses in employing the six-factor model of personality within the field. The main limitations with the study can be addressed by further studies within the same tradition. Meanwhile, however, these findings may sound a well-documented note of caution against advocacy for the use of the HEXACO in the field of clergy wellbeing research.

## Data Availability

Data are available from the corresponding author on reasonable request.

## Appendix 1

See Table

**Table 5:** HEXACO: scale properties

	*r*	%
*Honesty and humility* (*α* = .71)		
I wouldn’t use flattery to get a raise or promotion at work, even if I thought it would succeed	.30	73
If I knew that I could never get caught, I would be willing to steal a million dollars*	.28	4
Having a lot of money is not especially important to me	.39	72
I think that I am entitled to more respect than the average person is*	.43	18
If I want something from someone, I will laugh at that person’s worst jokes*	.44	6
I would never accept a bribe, even if it were very large	.33	72
I would get a lot of pleasure from owning expensive luxury goods*	.38	8
I want people to know that I am an important person of high status*	.42	11
I wouldn’t pretend to like someone just to get that person to do favours for me	.33	67
I’d be tempted to use counterfeit money, if I were sure I could get away with it*	.41	3
*Emotionality* (*α* = .56)		
I would feel afraid if I had to travel in bad weather conditions	.24	27
I sometimes can’t help worrying about little things	.17	41
When I suffer from a painful experience, I need someone to make me feel comfortable	.44	58
I feel like crying when I see other people crying	.29	44
When it comes to physical danger, I am very fearful	.35	40
I worry a lot less than most people do*	.15	17
I can handle difficult situations without needing emotional support from anyone else*	.22	39
I feel strong emotions when someone close to me is going away for a long time	.18	57
Even in an emergency I wouldn’t feel like panicking*	.26	45
I remain unemotional even in situations where most people get very sentimental*	.16	13
*Extraversion* (*α* = .66)		
I feel reasonably satisfied with myself overall	.25	79
I rarely express my opinions in group meetings*	.33	12
I prefer jobs that involve active social interaction to those that involve working alone	.37	60
On most days, I feel cheerful and optimistic	.40	77
I feel that I am an unpopular person*	.26	24
In social situations, I’m usually the one who makes the first move	.48	50
The first thing I always do in a new place is to make friends	.27	63
Most people are more upbeat and dynamic than I generally am*	.33	22
I sometimes feel that I am a worthless person*	.27	14
When I’m in a group of people, I’m often the one who speaks on behalf of the group	.31	35
*Agreeableness* (*α* = .46)		
I rarely hold a grudge, even against people who have badly wronged me	.27	63
People sometimes tell me that I am too critical of others*	.26	10
People sometimes tell me that I’m too stubborn*	.16	36
People think of me as someone who has a quick temper*	.31	9
My attitude toward people who have treated me badly is ‘forgive and forget’	.26	66
I tend to be lenient in judging other people	.08	55
I am usually quite flexible in my opinions when people disagree with me	.09	48
Most people tend to get angry more quickly than I do	.03	26
Even when people make a lot of mistakes, I rarely say anything negative	.22	33
When people tell me that I’m wrong, my first reaction is to argue with them*	.20	29
*Conscientiousness* (*α* = .73)		
I plan ahead and organise things, to avoid scrambling at the last minute	.43	75
I often push myself very hard when trying to achieve a goal	.39	87
When working on something I don’t pay much attention to small details*	.44	17
I make decisions based on the feeling of the moment rather than on careful thought*	.35	18
When working, I sometimes have difficulties due to being disorganised*	.45	24
I do only the minimum amount of work needed to get by*	.40	5
I always try to be accurate in my work, even at the expense of time	.50	84
I make a lot of mistakes because I don’t think before I act*	.31	18
People often call me a perfectionist	.28	39
I prefer to do whatever comes to mind, rather than stick to a plan*	.40	14
*Openness to experience* (*α* = .63)		
I would be quite bored by a visit to an art gallery*	.42	9
I’m interested in learning about the history and politics of other countries	.30	71
I would enjoy creating a work of art, such as a novel, a song, or a painting	.42	71
I think that paying attention to radical ideas is a waste of time*	.02	33
If I had the opportunity, I would like to attend a classical music concert	.45	66
I’ve never really enjoyed looking through an encyclopaedia*	.39	11
People have often told me that I have a good imagination	.21	61
I like people who have unconventional views	.03	51
I don’t think of myself as the artistic or creative type*	.39	24
I find it boring to discuss philosophy*	.39	8

^*^This item has been reverse coded to compute the correlations, but not the percentage endorsement

5 here.

## Appendix 2

See Table

**Table 6:** Francis burnout inventory: scale properties

	*r*	%
*Scale of emotional exhaustion in ministry* (*α* = .82)		
I feel drained by fulfilling my ministry roles	.40	15
Fatigue and irritation are part of my daily experience	.60	16
I am invaded by sadness I can’t explain	.62	7
I am feeling negative or cynical about the people with whom I work	.49	6
I always have enthusiasm for my work*	.35	71
My humour has a cynical and biting tone	.56	5
I find myself spending less and less time with those among whom I minister	.47	9
I have been discouraged by the lack of personal support for me here	.41	19
I find myself frustrated in my attempts to accomplish tasks important to me	.52	15
I am less patient with those among whom I minister than I used to be	.51	14
I am becoming less flexible in my dealings with those among whom I minister	.49	13
*Satisfaction in ministry scale* (*α* = .85)		
I have accomplished many worthwhile things in my current ministry	.45	75
I gain a lot of personal satisfaction from working with people in my current ministry	.53	81
I deal very effectively with the problems of the people in my current ministry	.42	71
I can easily understand how those among whom I minister feel about things	.46	73
I feel very positive about my current ministry	.63	82
I feel that my pastoral ministry has a positive influence on people’s lives	.58	85
I feel that my teaching ministry has a positive influence on people’s faith	.61	85
I feel that my ministry is really appreciated by people	.47	74
I am really glad that I entered the ministry	.61	89
The ministry here gives real purpose and meaning to my life	.53	92
I gain a lot of personal satisfaction from fulfilling my ministry roles	.60	86

^*^This item has been reverse coded to compute the correlations, but not the percentage endorsement

*r* correlation between individual items and the sum of the remaining items

6 here.
